# Neonatal and maternal outcomes of successful manual rotation to correct malposition of the fetal head; A retrospective and prospective observational study

**DOI:** 10.1371/journal.pone.0176861

**Published:** 2017-05-10

**Authors:** Nicola Tempest, Naomi McGuinness, Steven Lane, Dharani K. Hapangama

**Affiliations:** 1 Liverpool Women’s Hospital NHS Foundation Trust, Liverpool, United Kingdom; 2 Department of Women’s and Children’s Health, Institute of Translational Medicine, University of Liverpool, Liverpool, United Kingdom; 3 Department of Biostatistics, Institute of Translational Medicine, University of Liverpool, Liverpool, United Kingdom; Indiana University School of Medicine, UNITED STATES

## Abstract

**Objective:**

To evaluate the neonatal and maternal outcomes associated with successful operative vaginal births assisted by manual rotation.

**Design:**

Prospective and retrospective observational study.

**Setting:**

Delivery suite in a tertiary referral teaching hospital in England.

**Population:**

A cohort of 2,426 consecutive operative births, in the second stage of labour, complicated with malposition of the fetal head during 2006–2013.

**Methods:**

Outcomes of all births successfully assisted by manual rotation followed by direct traction instruments were compared with other methods of operative birth for fetal malposition in the second stage of labour (rotational ventouse, Kielland forceps and caesarean section).

**Main outcome measures:**

Associated neonatal outcomes (admission to the special care baby unit, low cord pH, low Apgar and shoulder dystocia) and maternal outcomes (massive obstetric haemorrhage (blood loss of >1500ml) and obstetric anal sphincter injury).

**Results:**

Births successfully assisted with manual rotation followed by direct traction instruments, resulted in 10% (36/346) of the babies being admitted to the Special Care Baby Unit, 4.9% (17/349) shoulder dystocia, 2% (7/349) massive obstetric haemorrhage and 1.7% (6/349) obstetric anal sphincter injury, similar to other methods of rotational births.

**Conclusions:**

Adverse neonatal and maternal outcomes associated with successful manual rotations followed by direct traction instruments were comparable to traditional methods of operative births. There is an urgent need to standardise the practice (guidance, training) and documentation of manual rotation followed by direct traction instrumental deliveries that will enable assessment of its efficacy and the absolute safety in achieving a vaginal birth.

## Introduction

Malposition of the presenting fetal head is defined as any position other than occiput anterior (eg. occipito-transverse (OT) or occipito-posterior (OP)) and is the commonest indication for caesarean section (CS) in the second stage of labour [1]. There is a severe lack of contemporary evidence from randomised trials to inform best practice for assisted operative birth when malposition complicates the second stage of labour [2,3]. The American College of Obstetrics and Gynecology advises that operative vaginal birth in the second stage of labour by experienced, proficiently trained physicians should be considered as a safe and acceptable alternative to CS whilst highlighting the need for training, and ongoing maintenance of practical skills in this context [4].

Manual rotation (MROT) where the fetal head is rotated and corrected by the operators’ hand, prior to completing the vaginal birth with direct traction forceps or non-rotational ventouse is increasingly being used in the second stage of labours complicated with fetal malposition where a prior requirement for expedited delivery exists [5]. Most regulatory authorities in the western world, either endorse the routine use or initial consideration of MROT followed by direct traction instruments in such labours prior to embarking on a traditional rotational instrumental birth or CS [4,6]. However, outcome data on morbidity and mortality associated with MROT (which includes subsequent direct traction instruments to complete birth as a single entity) in this context are scarce [7]. Some authors refer to anecdotal evidence of efficacy and safety [8], whilst others seem to rely on their personal perception that MROT is a safe procedure [9].

There are many described methods of MROT [8–10] and reports suggest that correcting malposition of the fetal head with MROT even without immediately completing the delivery with the use of a direct traction instrument may decrease the rates of both instrumental births and CS [9,11–14]. A recent Cochrane systematic review highlighted the need for further appropriately designed trials to determine the efficacy of MROT [9]. Compared with the explicit and formal guidance provided by the regulatory authorities on performing, documenting and training for other operative methods of births [2,6,8], similar guidance is not available for MROT. This, when combined with the different methods described to complete a MROT [8–10] may result in a potentially highly unregulated procedure.

Considering this paucity of evidence and the absence of guidance, our primary aim was to test the hypothesis that the successful use of MROT (a method which includes the immediate use of direct traction forceps/ventouse to complete the delivery) would be associated with comparable neonatal and maternal outcomes to all other established methods of operative births that can be employed to deliver a malpositioned fetus in the second stage of labour. The other operative methods considered included rotational ventouse (RV), Kielland forceps (KF) and primary emergency caesarean section in the second stage of labour without a trial of instrumental birth (pEMCS).

## Materials and methods

Liverpool Women's Hospital (LWH) has approximately 8,500 births per annum and is a tertiary referral centre with level three neonatal intensive care facilities. The LWH labour ward is directly supervised by a senior registrar in obstetrics (with at least 6–7 years of specialist training) 24 hours a day. In addition, there is at least one junior registrar (with a minimum of 3 years of specialist training) and an early career trainee with less than 3 years of specialist training. Throughout the study period, there was 90 hours per week of direct consultant presence, whilst indirect consultant supervision (consultant contactable by telephone for advice and to attend on demand as required) was available at all other times. In addition, local guidelines mandated that all births for malposition in the second stage of labour should be directly supervised or conducted by an experienced obstetrician (senior registrar with at least 6–7 years of specialist training in obstetrics or a consultant). All senior trainees were initially directly supervised in performing operative rotational vaginal births and subsequently deemed competent by consultant staff before being permitted for independent practice or to supervise other trainees.

For the purpose of our study, MROT births were defined as births where malposition was identified in the second stage of labour and MROT was employed to correct malposition followed by the immediate use of either direct traction forceps or non-rotational ventouse to complete the vaginal birth. The deliveries were assisted by direct traction forceps or non-rotational ventouse since expedition of birth had been deemed necessary due to fetal distress or delay.

Neonatal and maternal outcome data in all successful MROT births and all other successful traditional operative methods of assisting birth for a malpositioned fetal head in the second stage (RV, KF and pEMCS) were collected during the period from the 2^nd^ November 2006-31^st^ of August 2013 and reported as per STROBE guidelines [15]. All assisted rotational vaginal operative births and pEMCS (n = 2058) were identified retrospectively from the hospital database for the 60 month period from the 2^nd^ of November 2006 to the 31^st^ of August 2012, and reviewed. From the 1^st^ of September 2012 to the 31^st^ of august 2013 we prospectively collected outcome data, using a paper based proforma, (n = 368). After each delivery complicated by malposition at full dilatation, the operating surgeon completed a paper based proforma during the prospective part of the audit. These proformas were collected by the clinical team completing the study and the data (no patient identifiable data was collected or retained) was input into an EXCEL file. Data from the retrospective database or paper-based proformas were verified against the information in the computerised hospital notes system used at LWH (Meditech), the paper based hospital clinical records, the LWH risk management database, transfusion database, postnatal perineal clinic data, neonatal computerised notes database (BADGER; for all neonatal outcomes) before tabulating. When collecting demographic data, we considered the factors that may have influenced both the choice and the potential outcomes of the method employed to assist birth. For example, when the indication to assist birth is a diagnosis of potential fetal compromise, it may affect neonatal outcomes, as well as the decision making process regarding the method of birth. The operator seniority is expected to influence failure rates and outcomes; and there is prior evidence that epidural analgesia during labour increases vaginal operative birth rates [16]. A prolonged second stage of labour, has been associated with reduced umbilical cord pH values and Apgar scores [17,18]. Birth weight impacts on success rates of operative vaginal birth [19], in addition to increasing the risk for other outcome measures such as shoulder dystocia, obstetric anal sphincter injury (OASI) and massive obstetric haemorrhage.

Maternal and neonatal outcomes were chosen for their important clinical relevance, associated morbidity for the mother and baby and cost implications for the health care provider (National Health Service) and society. Admission to special care baby unit (SCBU), incidence of babies with cord pH <7.1 at birth, Apgar score <5 at 5 minutes, and the incidence of shoulder dystocia (defined as a vaginal cephalic delivery that requires additional obstetric manoeuvres to deliver the fetus after the head has delivered and gentle traction has failed [20]) were the neonatal outcomes collected. Maternal outcome variables collected were; massive obstetric haemorrhage (blood loss of >1500 ml, estimated or weighed following delivery) and OASI (3^rd^ or 4^th^ degree tear).

The finalised dataset of 2,426 singleton births at 36 weeks or more gestation was entered into excel spreadsheets (all patient identifiable data were excluded) and migrated into SPSS version 22 for analysis. Ethical approval was not required for these audits, (no patient identifiable data was collected) for which approval was granted by the directorate audit committee at LWH. No formal sample size was calculated as we collected data on all cases over a specified time period. Continuous variables were reported using means and standard deviations, categorical variables were reported using counts and percentages. Between group differences were initially assessed using analysis of variance and chi-squared tests. If a difference was detected between the four groups, pairwise post-hoc tests, with Bonferroni correction, were used to identify which groups were responsible for the differences.

## Results

**Fig 1:**
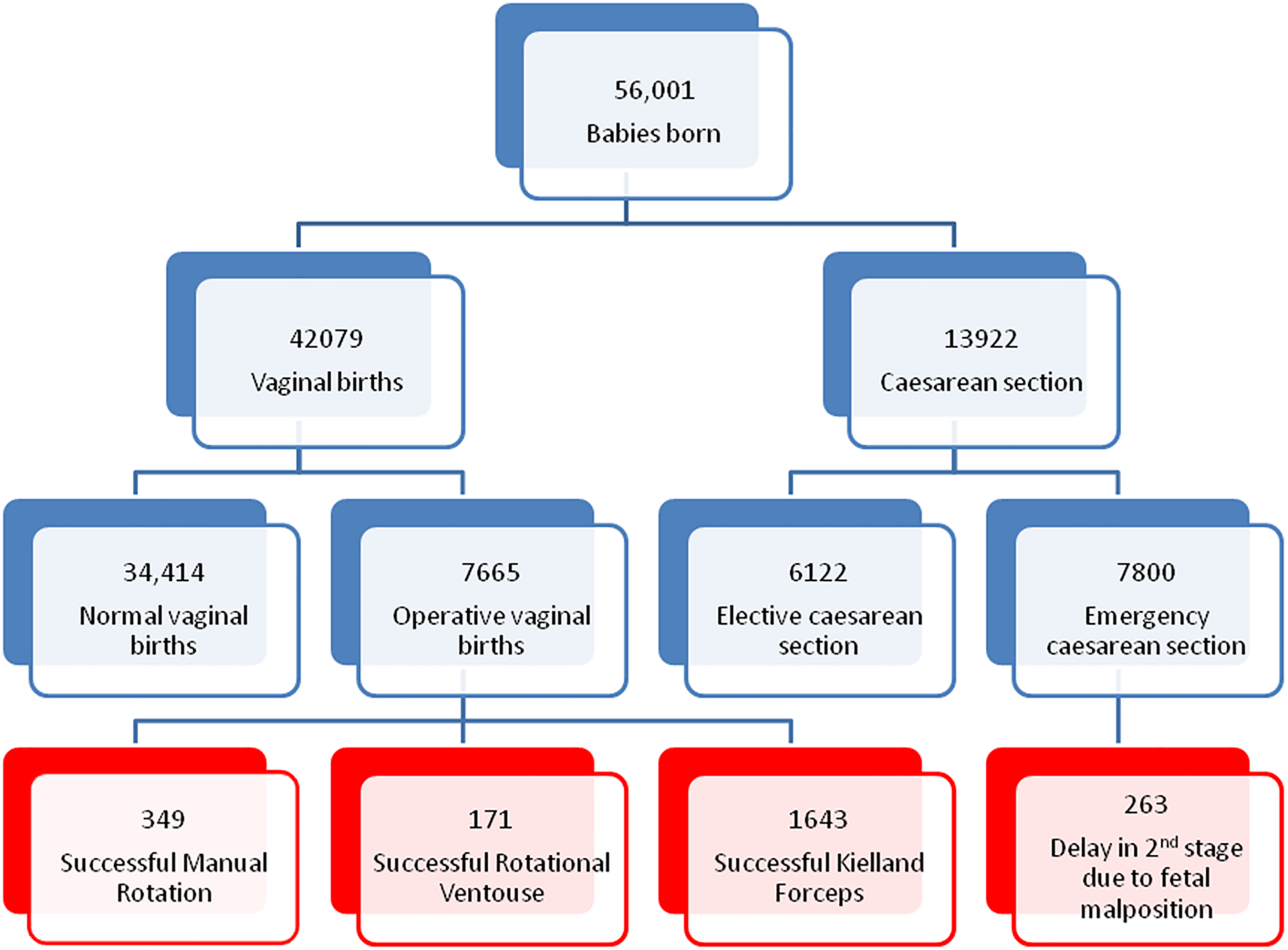
A Flowchart demonstrating how our final data set was captured.

There were 56,001 babies born at the LWH from the 2^nd^ Nov 2006 until the 31^st^ Aug 2013, of these, 42,079 were vaginal births, 34,414 were normal spontaneous vaginal births, not requiring assistance (61.5%) and 7,665 vaginal births required operative assistance (13.7%). 6122 births were by planned elective caesarean sections (10.9%); 7800 births were by emergency caesarean section (13.9%), 790 of those were performed at full dilatation (1.4%); out of which 263 were performed for the primary indication of delay in second stage of labour due to fetal malposition without a trial of instrumental birth (pEMCS). There were 2,426 consecutive full term singleton cephalic births between the 2^nd^ of Nov 2006 and the 31^st^ of Aug 2013 with malposition of the fetal head during the 2^nd^ stage of labour leading to a successful rotational delivery or pEMCS. Our final dataset included 349 successful MROT (0.62%) 171 successful RV (0.31%), 1,643 successful KF (2.93%) and 263 pEMCS (0.47%) (Fig 1).

The maternal age, induction of labour and seniority of doctor did not differ among the patient groups (Table 1).

**Table 1:** Demographics.

Variable	MROT (n = 349)	RV (n = 171)	KF (n = 1643)	pEMCS (n = 263)	Significance
Age (yrs)^1^ mean (st. dev)	29.25 (5.73)	29.35 (5.97)	29.25 (5.78)	30.29 (5.96)	P = 0.06
BMI^1^ mean (st.dev)	24.89 (4.79)	24.48 (5.18)	25.26 (4.89)	26.83 (6.27)	P<0.001
Multiparity^2^ N (%)	56(16.0)	49(28.8)	289 (17.6)	49(18.6)	P = 0.002
Induction^2^ N (%)	115 (33.0)	59 (34.5)	573 (34.9)	92 (35.0)	P = 0.92
Epidural^2^ N (%)	169/348 (48.6)	89/171 (49.7)	833/1642 (50.7)	59/263 (22.4)	P<0.001
Duration 1^st^ stage^1^* Mean (st. dev)	530.87 (306.48)	442.62 (269.73)	556.71 (322.64)	574.45 (291.34)	P<0.001
Duration 2^nd^ stage^1^* Mean (st. dev)	160.70 (141.29)	119.51 (77.33)	194.27 (77.33)	213.84 (83.91)	P = 0.001
Fetal compromise as indication^2^ N (%)	101 (28.9)	88 (51.5)	264 (16.1)	33 (12.5)	P<0.001
Operator ST6 or above^2^ N (%)	203/346 (58.7)	99/171 (58.2)	1049/1639 (63.5)	157/259 (60.6)	P = 0.23
Birth Weight^1^ Mean (st. dev)	3419.05 (545.24)	3394.71 (508.22)	3542.54 (499.94)	3682.62 (559.50)	P<0.001

^1^ ANOVA.

^2^ Chi-squared test.

* Data incomplete (343 and 345 MROT, 168 and 170 RV, 1638 and 1626 KF, 185 and 185 pEMCS).

However, there were statistically significant differences between the study groups in the following variables; BMI, multi-parity, rate of epidural, duration of first and second stage of labour, evidence of coexisting fetal compromise as a secondary indication for birth and birth weight. Considering neonatal outcomes, admission to the SCBU after MROT was not significantly different from that of other modes of birth (Table 2). Similar to the rates observed with KF births (9.9%, 163 out of 1,643), MROT was associated with a 10.3% (36/349) rate of admission to the SCBU. The babies born by pEMCS carried the highest rate of admission to the SCBU at 12.5% (33 out of 263 births) whilst RV use was associated with a much lower admission rate of 6.4% (11 of 171 births).

**Table 2:** Neonatal and maternal outcomes.

Variable	MROT (n = 349)	RV (n = 171)	KF (n = 1643)	pEMCS (n = 263)	Significance
Neonatal admission N(%)^1^	36/349 (10.3)	11/171 (6.4)	163/1643 (9.9)	33/263 (12.5)	P = 0.23
Shoulder Dystocia N(%)^1^	17/349 (4.9)	6/171 (3.5)	95/1643 (5.8)	NA	P = 0.24
Apgar<5 at 5 mins N(%)^1^	1/348 (0.3)	0/170 (0)	6/1640 (0.4)	3/263 (1.1)	
ApH <7.1 N(%)^1^	14/296 (4.7)	9/135 (6.7)	89/1401 (6.4)	13/235 (5.5)	P = 0.72
EBL >1.5L N(%)^1^	7/349 (2.0)	4/171 (2.3)	38/1642 (2.3)	13/263 (4.9)	P = 0.08
Sphincter injury N(%)^1^	6/349 (1.7)	1/171 (0.6)	48/1642 (2.9)	NA	P = 0.10

^1^ Chi-squared.

Only a very small number of babies in our dataset were born with Apgar scores less than 5 at 5 minutes across all of the groups, and no statistical differences were noted. For example, only one baby had an Apgar recorded of less than 5 at 5 minutes (0.3%) in the MROT group. This figure compared favourably with babies born in the KF group (0.4%, 6 of 1640) and in our cohort there were no babies born with an Apgar score of less than 5 at 5 minutes in the RV group. Compared to the rotational vaginal operative births, 3 out of 263 (1.1%) of the pEMCS births were associated with babies having a low Apgar score at 5 minutes.

The rate of umbilical arterial cord pH <7.1, was also comparable in all groups and MROT had the lowest rates at 4.7%, (14 of 296 babies), 5.5%, (13 of 235 babies) in the pEMCS group; 6.4% (89 of 1401 babies) in the KF group; and RV births were associated with 6.7% (9 of 135 babies) low umbilical arterial pH.

The rate of shoulder dystocia following MROT was similar to the rates associated with either RV or KF. The overall rate of shoulder dystocia with MROT was 4.9% (17 of 349), whilst it was 3.5% (6 of 171) with RV, and conversely KF births were associated with a slightly higher rate of 5.8% (95 of 1643).

Out of the 349 births assisted with MROT, 7 mothers (2%) had a massive obstetric haemorrhage (Table 2). This compares with the rates observed in both RV and KF groups, which in our cohort also carried a similar risk of massive obstetric haemorrhage, at 2.3%. Conversely, the 263 women who underwent pEMCS had an approximately doubled rate of massive obstetric haemorrhage at 4.9% yet this did not reach a statistical significance.

In our patient cohort, 55 out of all 2,162 women who experienced a successful vaginal operative birth had an OASI with an overall rate of 2.5%. The incidence of OASI specific to the MROT group was 1.7% (6 of 349), higher than that associated with RV of 0.6% (1 of 171 births) and although not statistically different, lower than that of the KF group at 2.9% (48 of 1642).

## Discussion

### Main findings

Our data suggests that successful use of MROT is associated with comparable neonatal and maternal complication rates to all other established methods of rotational operative births suitable for a malpositioned fetal head. There were no statistically different neonatal or maternal outcomes between all four modes of birth, confirming that all methods are equally acceptable to be utilised in clinical practise.

Considering most demographic features studied, MROT offered an intermediate alternative to both RV and KF; in that, comparing with RV, MROT was the choice in nulliparous women with slightly longer labours (1^st^ and 2^nd^ stage), whilst, RV was preferred when there was evidence of fetal compromise. The opposite was true for the comparison between MROT and KF. Finally, when compared with the pEMCS group, MROT was the method of choice for women with epidurals and a higher rate of fetal compromise but pEMCS appears to be preferentially used when a woman had a higher BMI, a longer second stage or a larger baby. Furthermore, factors that are well known to be associated with an increased risk of caesarean section in general such as higher BMI, birth weight and age were also associated with 2^nd^ stage pEMCS group compared with the operative deliveries.

If MROT is to be utilised as a formal method of rotational vaginal birth, it must be comparable in neonatal and maternal safety parameters with all other available modes of operative births. When we considered subtle trends in safety data, similar to the demographic features, MROT occupied an equidistant position between RV and KF, in shoulder dystocia and OASI, where higher rates were seen with KF. MROT however was associated with slightly higher numbers of neonatal admissions to SCBU but had similar rates of massive obstetric haemorrhage compared with both KF and RV. Agreeing with previous publications [21,22] pEMCS carried the highest risk of massive obstetric haemorrhage and rates of neonatal SCBU admission. Therefore, although these trends are not statistically significant, we have not observed convincing data to conclude the perceived superiority of MROT use for neonatal or maternal safety against other methods of rotational births. Taken together our data suggest fetal and maternal outcomes associated with successful MROT were comparable with all other methods of rotational operative births at best (when successful), when malposition complicates the second stage of labour.

### Strengths and limitations

This is the largest reported series of consecutive operative rotational vaginal births successfully assisted by MROT to be reported from a single, busy, modern, tertiary referral hospital. Although our study was primarily designed to describe the outcomes of births assisted by MROT, our case series of rotational operative vaginal births is also the largest consecutive series ever published to include all available methods when malposition complicates the second stage of labour. Despite the above, this study was not powered to examine extremely rare cases of outcomes such as neonatal death.

Since in our unit, all obstetricians practising MROT were proficient in the use of at least one other (some all) methods of rotational operative births, our data highlight the specific demographic features that may lead clinicians to favour the choice of MROT over other methods of vaginal operative births.

The main drawback of our study is the retrospective nature of data collection employed during the first part of the study. In our unit, routine documentation of successful MROTs was similar to other operative rotational vaginal births (therefore we consider that almost all successful attempts with MROT were documented), yet failed attempts were not consistently documented. Consequently, reliable data retrieval was only possible on unsuccessful MROT during the prospective study. A similar practise of inconsistent documentation is expected to prevail in other units; therefore, our data underscores the urgent need to standardise documentation for this high risk obstetrics practise. Furthermore, the inconsistent documentation of unsuccessful MROT may also be a major obstacle in assessing the “true” effectiveness and safety of any other operative vaginal birth. We would add a caveat, that the outcomes we report for successful MROTs may be different in births that are unsuccessfully assisted by MROT.

Our study examined MROT as a single entity, defined as the rotation of the fetal head to correct malposition by hand and the immediate subsequent application of direct traction forceps/ventouse to complete birth. Our study did not intend to examine the different outcomes associated with the non-rotational, direct traction instruments used after the MROT. There are many previous publications describing the outcomes associated with non-rotational direct traction forceps vs. ventouse and future well powered studies can examine the influence of post MROT traction instruments on the clinical outcomes.

A further deficiency of this study is the lack of information on the subjective but important information on the station of fetal head, presence of caput, strength of uterine contractions and findings of the abdominal examination. This information should be collected in future prospective multicentre cohort studies to assess the best method of birth for malposition.

### Interpretation

Our data show that the use of MROT is associated with a low incidence of complications for both the infant and the woman and compares favourably with other techniques. However, these complications need to be carefully considered when another consecutive rotational operative vaginal delivery method is attempted in instances when MROT is unsuccessful [6]. Our large dataset provide information on outcomes associated with the general use of the method, however these observations will still be subjected to selection bias and the influence of skills of highly selected individuals. Our findings are observed within a specific setting where all operative techniques were either performed or directly/indirectly supervised by a group of UK consultants, most whole time obstetricians providing ongoing support and training for trainees. The low rate of sphincter damage we observe in our dataset compared with previous reports may be another consequence of this degree of senior supervision [2].

### Conclusion–practical and research recommendations

We present here the largest series of consecutive operative rotational vaginal births successfully assisted by MROT, suggesting that successful use of MROT is associated with comparable neonatal and maternal complication rates to all other established methods of operative births suitable for a malpositioned fetal head.

MROT is increasingly being employed for assisting births throughout the world, yet there is a lack of published data on safety and efficacy. Our data highlights that MROT has a role in the management of malposition at full dilatation, with comparable outcomes to traditional methods such as RV and KF. It also provides the initial evidence to encourage obstetricians and the regulating authorities to formally recognise MROT, as a high risk, practical obstetrics procedure similar to the other methods of traditional rotational operative deliveries, and formulate the fundamentally essential, yet presently non-existing guidance (including documentation), training and governance process for MROT which in parallel with other traditional methods requires the highest level of skill in acute obstetric practise. Clinicians and units that encourage the use of MROT have a responsibility to collect morbidity data which allows women and clinicians to make informed choices.

**Fig 2:**
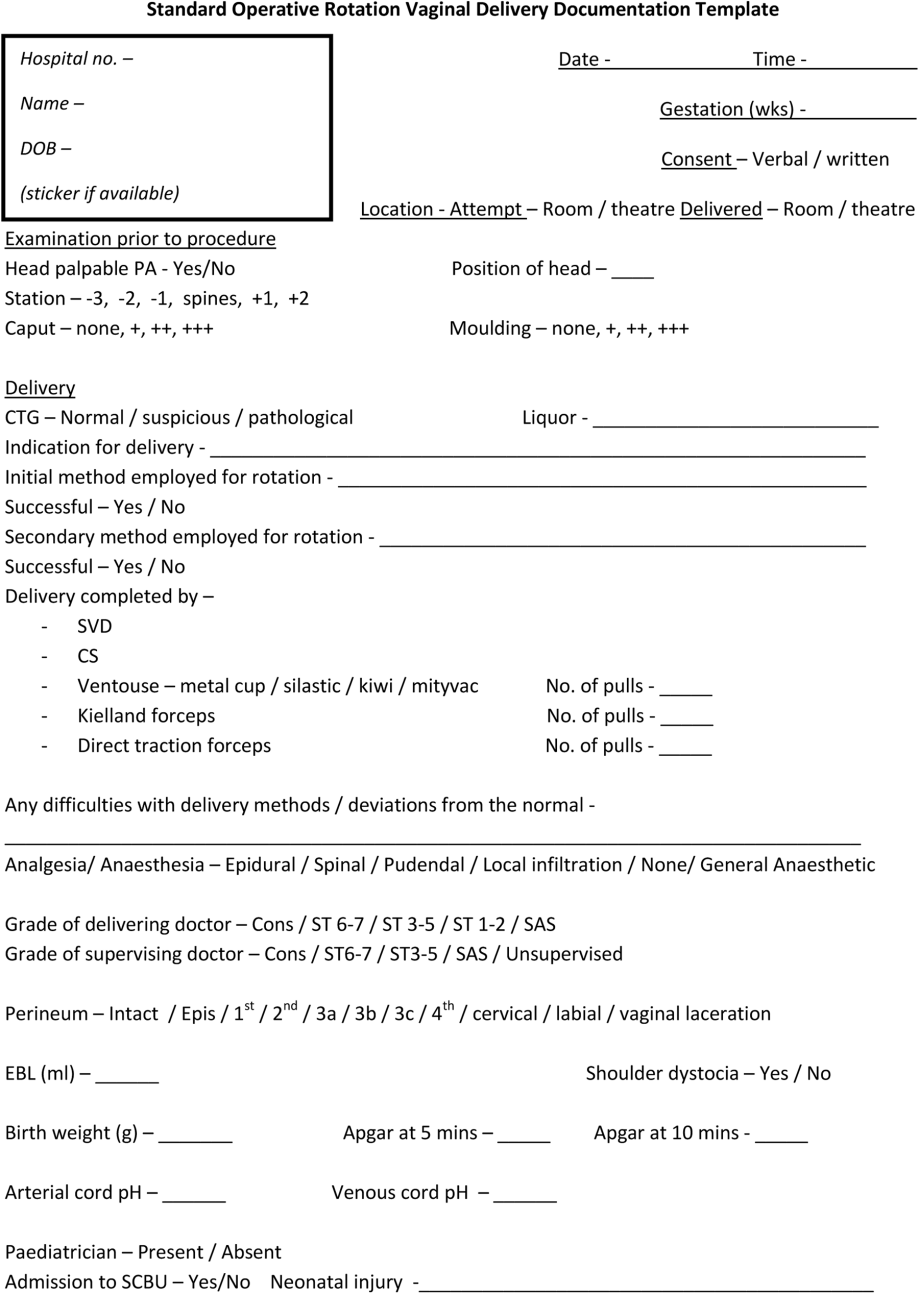
A Sample standard operative rotation vaginal delivery documentation template. Proposed documentation proforma to record essential data that can be used in all operative methods to expedite delivery in second stage labours complicated with fetal malposition.

Formal documentation of MROT is necessary to assess the true efficacy of all rotational delivery methods, and more importantly to assess the complication rates associated with unsuccessful attempts of using all methods. We propose that standardised documentation (Fig 2) will not only facilitate collating data from various studies as well as reducing bias in any future prospective trials.

We conclude that our study highlights the urgent need to standardise the practice and documentation of operative births when malposition of the fetal head complicates the second stage of labour. A national, regulated prospective data collection of the outcomes of high-risk obstetric interventions including rotational births and EMCS in the second stage of labour is necessary and overdue. A reliable and standard system of reporting the efficacy and safety outcomes of these high risk births will ensure the maintenance of high standards hence quality assurance, service improvement, a benchmark for the production of formal training programmes and guidelines, assist greater public transparency and accountability in obstetric practise through a public reporting system similar to other surgical outcomes. We believe these essential changes will ensure babies and mothers receive the most appropriate and highest level of care when labour is complicated by malposition.

## References

[1] Tempest N, Navaratnam K, Hapangama DK. Does advanced operative obstetrics still have a place in contemporary practice? Curr Opin Obstet Gynecol. 2015;27(2):115–20. doi: 10.1097/GCO.0000000000000159 25692505 10.1097/GCO.0000000000000159

[2] RCOG. Operative vaginal delivery Green-top guidelines. London (UK): Royal College of Obstetricians and Gynaecologists; 2011.

[3] Majoko F, Gardener G. Trial of instrumental delivery in theatre versus immediate caesarean section for anticipated difficult assisted births. Cochrane Database Syst Rev 2012;17;10:CD005545. 10.1002/14651858.CD005545.pub3 PMC4171385 23076915

[4] American College of Obstetricians and Gynecologists (College), Society for Maternal-Fetal Medicine, Caughey AB, Cahill AG, Guise JM, Rouse DJ. Safe prevention of the primary cesarean delivery. Am J Obstet Gynecol. 2014;210(3):179–93. doi: 10.1016/j.ajog.2014.01.026 24565430 10.1016/j.ajog.2014.01.026

[5] Chinnock M, Robson S. An anonymous survey of registrar training in the use of Kjelland's forceps in Australia. Aust N Z J Obstet Gynaecol. 2009;49(5):515–6. doi: 10.1111/j.1479-828X.2009.01058.x 19780736 10.1111/j.1479-828X.2009.01058.x

[6] RANZCOG. Rotational Forceps. Victoria (Australia): Royal Australian and New Zealand College of Obstetricians and Gynaecologists; 2012.

[7] Bahl R, Van de Venne M, Macleod M, Strachan B, Murphy DJ. Maternal and neonatal morbidity in relation to the instrument used for mid-cavity rotational operative vaginal delivery: a prospective cohort study. BJOG. 2013;120(12):1526–32. doi: 10.1111/1471-0528.12398 23924292 10.1111/1471-0528.12398

[8] SOGC. Guidelines for operative vaginal birth. Ontario (Canada): Council of the Society of Obstetricians and Gynaecologists of Canada; 2004.

[9] Phipps H, de Vries B, Hyett J, Osborn DA. Prophylactic manual rotation for fetal malposition to reduce operative delivery. Cochrane Database Syst Rev 2014;22;12:CD009298. 10.1002/14651858.CD009298.pub2 PMC11032750 25532081

[10] Scott JR, Gibbs RS, Karlan BY, Haney AF. Danforth`s obstetrics and gynaecology, 9th ed. Philadelphia (PA): Lippincott Williams & Wilkins; 2003.

[11] Le Ray C, Deneux-Tharaux C, Khireddine I, Dreyfus M, Vardon D, Goffinet F. Manual rotation to decrease operative delivery in posterior or transverse positions. Obstet Gynecol. 2013;122(3):634–40. doi: 10.1097/AOG.0b013e3182a10e43 23921875 10.1097/AOG.0b013e3182a10e43

[12] Sen K, Sakamoto H, Nakabayashi Y, Takeda Y, Nakayama S, Adachi T et al Management of the occiput posterior presentation: a single institute experience. J Obstet Gynaecol Res. 2013;39(1):160–165. doi: 10.1111/j.1447-0756.2012.01935.x 22765887 10.1111/j.1447-0756.2012.01935.x

[13] Shaffer BL, Cheng YW, Vargas JE, Caughey AB. Manual rotation to reduce caesarean delivery in persistent occiput posterior or transverse position. J Matern Fetal Neonatal Med 2011;24(1):65–72. doi: 10.3109/14767051003710276 20350240 10.3109/14767051003710276

[14] Minas V, Khafizova L. A novel modification to manual rotation of the foetal head: making the manoeuvre safer. Arch Gynecol Obstet. 2013;287(4):825–6. doi: 10.1007/s00404-012-2570-5 23001367 10.1007/s00404-012-2570-5

[15] von Elm E, Altman DG, Egger M, Pocock SJ, Gøtzsche PC, Vandenbroucke JP, STROBE Initiative. The Strengthening the Reporting of Observational Studies in Epidemiology (STROBE) statement: guidelines for reporting observational studies. Journal of Clinical Epidemiology. 2008;61(4):344–349 doi: 10.1016/j.jclinepi.2007.11.008 18313558 10.1016/j.jclinepi.2007.11.008

[16] Anim-Somuah M, Smyth RM, Jones L. Epidural versus non-epidural or no analgesia in labour. Cochrane Database Syst Rev 2011; 7;12:CD000331. 10.1002/14651858.CD000331.pub3 22161362

[17] Li WH, Zhang HY, Ling Y, Jin S. Effect of prolonged second stage of labour on maternal and neonatal outcomes. Asian Pac J Trop Med. 2011;4:409–411. doi: 10.1016/S1995-7645(11)60114-4 21771687 10.1016/S1995-7645(11)60114-4

[18] Bleich AT, Alexander JM, McIntire DD, Leveno KJ. An analysis of second-stage of labour beyond 3 hours in nulliparous women. Am J Perinatol. 2012;29:217–222. 22644830 10.1055/s-0032-1314894

[19] Murphy DJ, Liebling RE, Verity L, Swingler R, Patel R. Early maternal and neonatal morbidity associated with operative delivery in second stage of labour: a cohort study. Lancet. 2001;358:1203–1207. doi: 10.1016/S0140-6736(01)06341-3 11675055 10.1016/S0140-6736(01)06341-3

[20] RCOG. Shoulder dystocia Green-top Guidelines. London (UK): Royal College of Obstetricians and Gynaecologists; 2012.

[21] Stock SJ, Josephs K, Farquharson S, Love C, Cooper SE, Kissack C et al Maternal and neonatal outcomes of successful Kielland's rotational forceps delivery. Obstet Gynecol. 2013;121(5):1032–9. doi: 10.1097/AOG.0b013e31828b72cb 23635740 10.1097/AOG.0b013e31828b72cb

[22] Tempest N, Hart A, Walkinshaw S, Hapangama DK. A re-evaluation of the role of rotational forceps: retrospective comparison of maternal and perinatal outcomes following different methods of birth for malposition in the second stage of labour. BJOG 2013;120(10): 1277**–**84. doi: 10.1111/1471-0528.12199 23906197 10.1111/1471-0528.12199

